# Developmental asynchrony might not reduce fitness in early life in painted turtles

**DOI:** 10.3389/fphys.2022.923912

**Published:** 2022-08-26

**Authors:** P. L. Colbert, R.-J. Spencer, F. J. Janzen

**Affiliations:** ^1^ SAb Biotherapeutics, Sioux Falls, SD, United States; ^2^ School of Science, Western Sydney University, Penrith, NSW, Australia; ^3^ Kellogg Biological Station, Departments of Fisheries & Wildlife and Integrative Biology, Michigan State University, Hickory Corners, MI, United States

**Keywords:** *Chrysemys picta*, embryonic development, hatching synchrony, hatching success, survival, predator avoidance

## Abstract

Synchronous hatching and emergence of turtles from nests may be adaptive in predator avoidance during dispersal. However, little is known about the phenotypic consequences of such synchrony or the generality of predator avoidance in driving the evolution of this trait. Colbert et al. (2010) found that less advanced embryos hatched early in the presence of more advanced sibs, sustaining a persistent reduction in neuromuscular function. In this study, we experimentally assessed the influence of such accelerated embryonic development on hatching success, winter survival, and survival during terrestrial dispersal from the nest. Although we predicted that shortened incubation periods would reduce survival, early-hatching individuals suffered no detectable fitness costs at any stage considered in this study. Incubation temperature did not affect hatching success, and offspring sex did not affect survival across treatment groups. Incubation regime influenced offspring body size and was negatively correlated with dispersal time, however, there was no effect on survival during winter or terrestrial dispersal. Lack of a detectable fitness cost in these key early-life stages associated with hatching synchrony is consistent with a single, predator avoidance origin for this trait and retention in *C. picta* and other derived turtles via phylogenetic inertia.

## Introduction

An understanding of factors that contribute to phenotypic expression during development and early life is central to studies of trait evolution. It is often during these stages that phenotypic variation resulting from genetic, parental, and environmental effects is subject most stringently to selection. In oviparous organisms, particularly those lacking nest attendance, the incubation environment is of singular importance as eggs may be subject to substantial variation in abiotic conditions during development. Thermal variation in particular can influence developmental rate, hatching success, and the sex, morphology, and behavior of offspring (e.g., Ratte, 1985; Deeming and Ferguson, 1991; Andrews, 2004). For turtles, which are characterized by near universal lack of nest attendance, synchronous hatching and its phenotypic and broader biological consequences reside at the interface of the effects of temperature, development, and selection.

Turtle nests are often flask-shaped with eggs deposited in several layers. As a result, thermal gradients can exist such that eggs near the top experience warmer incubation conditions than those near the bottom (Thompson, 1988; Maloney et al., 1990; Telemeco et al., 2016). Because developmental rate generally increases with increasing temperature (Thompson, 1997), asynchronous hatching and emergence of neonates is predicted. Yet synchronous hatching and/or emergence occur (Carr and Hirth, 1961; Balasz and Ross, 1974; Spencer et al., 2001; but see Standing et al., 1999; Houghton and Hays, 2001) and may have evolved in response to predation during dispersal from nests. Hatching synchrony facilitates physical escape from the nest (Carr and Hirth, 1961; Spencer et al., 2001) and may aid predator avoidance via swamping or the per capita dilution of predation risk (Arnold and Wassersug, 1978; Dehn, 1990; Santos et al., 2016; but see Tucker et al., 2008). However, questions remain as to how synchronous hatching occurs, whether the mechanism producing such synchrony generates additional phenotypic variation in offspring, and, if so, how selection acts upon that variation.

Hatching synchrony has been explored in at least five turtle species, occurring in four of them. Riley et al. (2020) detected synchronous hatching in spiny softshell turtles (*Apalone spinifera*) but not in northern map turtles (*Graptemys geographica*). Spencer et al. (2001), Colbert et al. (2010), and Field et al. (2021) investigated synchronous hatching experimentally by inducing developmental asynchrony among clutch mates of Murray River turtles (*Emydura macquarii*), painted turtles (*Chrysemys picta*), and loggerhead turtles (*Caretta caretta*), respectively. Synchrony occurred in all cases because less developed embryos hatched earlier than normal while more advanced sibs did not delay hatching. However, early-hatching *C. picta* exhibited poor righting ability compared to their sibs both at hatching and after overwintering in nests. For *E. macquarii*, *C. caretta*, and *A. spinifera*, the potential benefits of group formation may outweigh any individual developmental costs as neonates generally depart the nest within days after hatching. In contrast, *C. picta* (like *G. geographica*; Gibbons and Nelson, 1978) typically remain in the nest up to 9 months after hatching (Weisrock and Janzen, 1999; Murphy et al., 2020). Thus, early hatching with reduced neuromuscular function, even in the absence of metabolic compensation (McGlashan et al., 2018), is puzzling for *C. picta*. In this study, we asked whether this seemingly detrimental trait in painted turtles was accompanied by reduced fitness, focusing specifically on survival during three key early-life stages in this species: 1) hatching, 2) over-winter in the nest, and 3) terrestrial dispersal from nest to water. In brief, do early-hatching neonates suffer detectable survival-linked costs in early life?

## Materials and methods

The painted turtle (*C. picta*) is a common inhabitant of freshwater environments from coast to coast in the northern U.S. and southern Canada and into parts of the southern U.S. (Ernst and Lovich, 2009). At our study site, typically 10 eggs are deposited in relatively shallow terrestrial nests (ca. 9 cm; Morjan, 2003) within which vertical temperature differentials during embryonic development may span as much as 6°C (FJJ unpubl. data). Following hatching, most neonates remain in the nest throughout the winter and emerge the following spring (Weisrock and Janzen, 1999; Murphy et al., 2020). Cohorts most closely follow a Type III survivorship schedule, with high mortality through the first few years of life followed by low mortality near maturity (Iverson, 1991a; Frazer et al., 1991; Warner et al., 2016). As such, phenotypic traits that offer survival advantages early in life are of great importance (Iverson, 1991a, b; Spencer and Janzen, 2010). Eggs were collected from 24 newly constructed nests (<1 day old) at the Thomson Causeway, Thomson, Illinois, United States from 27 May through 1 June 2004. Upon collection, eggs were labeled according to clutch and egg number using a blunt HB pencil and placed in moist vermiculite for transport to the laboratory. Sixteen of these 24 clutches were part of a previous study of the phenotypic effects of hatching synchrony (Colbert et al., 2010). Evaluation of hatching synchrony involved two experiments; one that tested whether less advanced embryos truncated development time to “catch up” to more advanced sibs while the other assessed whether more advanced embryos could “wait” to hatch in the presence of less advanced sibs.

**FIGURE 1:**
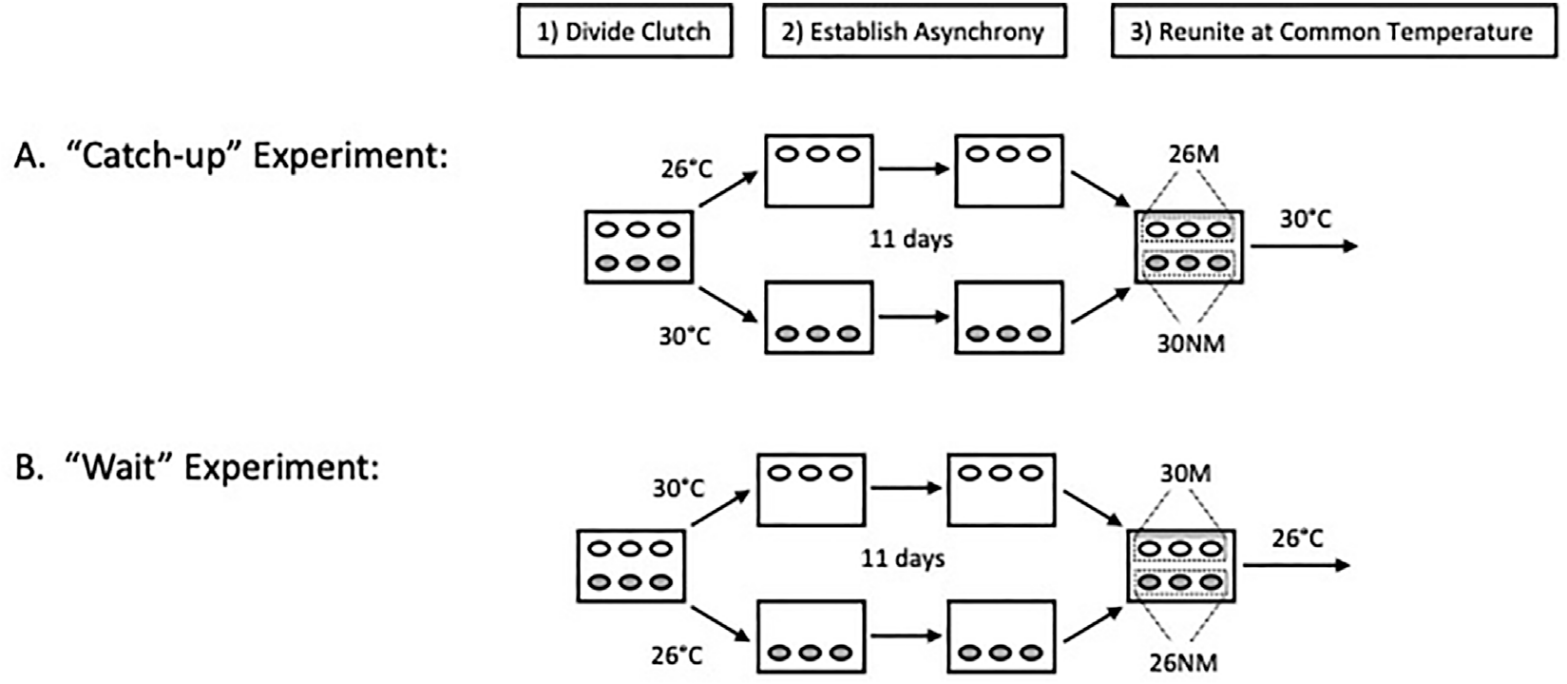
General incubation protocol (modified from Figure 2 of Colbert et al., 2010). The steps employed to establish developmental asynchrony and promote shortened incubation periods of less advanced embryos. Temperatures used (26 or 30°C) depended on the treatment and 11 days indicates the period over which asynchrony was established in the study.

Incubation regimes to establish developmental asynchrony among clutch-mates followed the general protocol of Spencer et al. (2001). Specifically, half clutches were incubated at either cool (26°C) or warm (30°C) temperatures for the first 11 days of incubation and were then reunited with clutch-mates at a common incubation temperature (Figure 1). Two treatment levels existed within each experiment based on group status (experimental or control [C]) and egg movement (moved [M] or not [NM]), resulting in eight distinct treatments (Table 1). The additional eight clutches were half of a replication of the synchrony experiment, the other half of which was lost due to incubator failure. All 24 clutches were treated in an identical manner and are therefore included, although sample sizes were unequal among treatments (Table 1). Throughout incubation, all egg boxes were maintained at a water potential of −150 kPa in vermiculite.

**TABLE 1:** Experimental groups, treatment designations, and clutch representation.

Experiment	Group	Initial Temp. (°C)	Moved	Final Temp. (°C)	Treatment	Clutches
	Experimental	26	No	26	*26NM*	8
Catch-up	Experimental	30	Yes	26	30M	8
	Control	26	No	26	26CNM	7
	Control	26	Yes	26	26CM	7
	Experimental	30	No	30	30NM	4
Wait	Experimental	26	Yes	30	*26M*	4
	Control	30	No	30	30CNM	5
	Control	30	Yes	30	30CM	5

Treatment abbreviations reflect the initial incubation temperature and group and movement status. Thus, 26CNM implies that the eggs were initially incubated at 26°C, belonged to a control group (C), and were not moved (NM). Similarly, 30M implies that the eggs were initially incubated at 30°C, belonged to an experimental group (lacks the control group designation, C), and were moved (M) to their sibs’ container at 26°C. The clutches column shows the number of half clutches assigned to each treatment. Experimental and control groups within each experiment have the same number of clutches because clutches were split between them. Each half clutch consisted of three eggs, yielding sample sizes ranging from 12 (30NM and 26M) to 24 (26NM and 30M). Groups known (26NM) or suspected (26M) to have hatched early and that exhibited reduced neuromuscular function in the laboratory (Colbert et al., 2010) are italicized.

Beginning at day 40 of incubation, eggs were visually inspected for signs of pipping (the initial breaking of the egg shell by the caruncle) at least three times daily. Neonates that extricated themselves from their eggshell were considered to have hatched successfully. Once fully hatched, individuals were removed from their containers and their righting times measured as a gauge of neuromuscular function. In this procedure, animals are placed on their carapace and the time required to right themselves (i.e., flip over) is recorded. Freedberg et al. (2001, 2004) note that hatchlings are highly motivated to perform this task and righting ability may be important to survival during terrestrial dispersal by reducing the risks of desiccation and predation (Burger, 1976; Steyermark and Spotila, 2001a; Delmas et al., 2007; but see Mitchell et al., 2016). Following righting trials, hatchlings were given a unique combination of notches in their marginal scutes and transferred to clutch-specific plastic cups containing a moist paper towel in preparation for over-winter torpor.

Over the winter months, temperatures in dark environmental chambers were maintained at approximately 5°C and cups were hydrated weekly with distilled water to prevent desiccation of hatchlings. Beginning in the second week of April 2005, chamber temperatures were slowly raised to 18°C (ca. 2°C/d for 7 days). Neonates were removed from the chambers and maintained at 22°C for 48 h before righting trials were again performed and morphological measurements taken (results reported in Colbert et al., 2010). We measured carapace length (CL; measured as the distance from the nuchal scute along the midline to the inter-marginal notch) to the nearest 0.1 mm using a dial caliper and body mass to the nearest 0.01 g using an electronic balance. Neonates that were alive at this point were recorded as having survived through the winter. For about 4 weeks, these turtles were maintained in plastic containers (60 cm × 30 cm x 15 cm) filled with de-chlorinated tap water to a depth of about 8 cm and containing several basking platforms under 12:12 lighting. Turtles were fed a diet of bloodworms and Reptomin^®^ *ad libitum*, and containers were cleaned twice weekly.

The neonatal dispersal experiment was conducted in the Upper Mississippi River National Fish and Wildlife Refuge in Carroll County, Illinois adjacent to the nesting area. The release site was on an open, west-facing slope of sand prairie where painted turtles are known to nest (Paitz et al., 2007). Vegetation in the immediate area of the experiment included needlegrass (*Stipa* sp.), Ohio spiderwort (*Tradescantia ohiensis*), prickly pear cactus (*Opuntia humifusa*), red cedar (*Juniperus virginiana*), and skunkbrush (*Rhus aromatica*). Kolbe and Janzen (2002a) and Warner and Mitchell (2013) provide further description of the study area.

A drift fence was constructed along the base of the slope near the east shore of the Mississippi River. The drift fence was comprised of 30-cm high aluminum flashing entrenched 10 cm in the soil and arranged as a semi-circle with a 13-m radius. Along the fence, seven numbered plastic cylinders (i.e., pits; 10 cm diameter × 25 cm deep) were buried flush with the ground, abutting the fence at 7-m intervals. A semi-circular design was used to reduce the chance of turtles escaping; recording which pits captured turtles allowed us to determine the likelihood that such an event might have occurred (see below). Six naturalistic overwinter nest cavities, or nesticles (Colbert, 2006), were created 10 cm apart at the center of the semi-circle and a brown tarp was pinned down over their back edges to leave a small opening through which neonates could emerge. Although 13 m is below the overall mean nest distance from water at our study site (Harms et al., 2005), it is not atypical (Kolbe and Janzen, 2001). The experiment was initiated at 1025 h on 25 May 2005 when 19-20 randomly chosen turtles were placed into each nesticle. This date falls within the range when neonatal *Chrysemys* typically disperse from nests in this area (Murphy et al., 2020), and at an hour when emergence is known to occur in related turtle species (Tucker, 1997).

Following release, the fence was checked at 1510 and 1950 h, and on each subsequent day at approximately 0700, 1300, and 1900 h until 0700 h on 7 June. Observers noted the presence of potential predators during each visit and the sand surrounding each pit was checked for tracks and subsequently brushed smooth. Neonates found along the fence or in pits were recovered and their identity and location (i.e., pit number 1-7) recorded. All turtles recovered alive at the fence were considered to have survived terrestrial dispersal. These animals were subsequently given a unique combination of toe clips and released in the water near the nest sites from which the eggs were collected.

Logistic regression was used to determine whether incubation treatment significantly influenced hatching success, over-winter survival, and survival during terrestrial dispersal. For the purposes of this study, the groups of primary interest were those known (26NM) or suspected (26M) to have hatched early (see definitions in Table 1) at a cost to neuromuscular function (Colbert et al., 2010). We expected that premature hatching would result in reduced hatching success relative to sibs and/or controls. In addition, we hypothesized that underdeveloped hatchlings may be more sensitive to temperature extremes and therefore suffer higher winter mortality. During terrestrial dispersal, we predicted that the developmental costs sustained by hatching early would increase the time required for individuals to migrate. Increased exposure to the rigors of the terrestrial environment (e.g., predation and desiccation) would, in turn, cause higher mortality in these treatments. Hence, in regression analyses of all three early-life stages, we set up the following contrasts concerning treatment effects on survival

### H_A_: 26NM < 26CNM, 30M

### H_A_: 26M < 30NM

A general linear model was used to test for treatment effects on the time required for individuals to reach the fence and evaluate the proposed exposure time mechanism for any reduced survival during terrestrial dispersal. Time was measured from the point of release until recapture to the nearest 0.25 days. Individuals recaptured on the first day were scored as reaching the fence in 0.25 days. On subsequent days, recapture times were recorded as composites of the day of capture relative to the release date and the time of day as follows: 0.25 (morning check), 0.5 (afternoon check), or 0.75 (evening check). Individuals with reduced neuromuscular performance were expected to take longer to arrive at the fence than their sibs and/or controls. Therefore, we set up the following contrasts concerning treatment effects on time to recapture:

### H_A_: 26NM > 26CNM, 30M

### H_A_: 26M > 30NM

*Chrysemys picta* exhibits temperature-dependent sex determination in which males are produced at cooler temperatures (typically <28.5 C) and females at warmer temperatures (Carter et al., 2019). In this study, temperature switches were performed prior to the temperature-sensitive period of sex determination (about the middle-third of incubation; Janzen and Paukstis, 1991), thus no treatment-specific comparisons are made between sexes. We evaluated the influence of sex on hatching success, survival, and time until recapture separately using logistic regression, but without any *a priori* predictions of its effect.

Many studies have documented that “bigger is better” during terrestrial dispersal from nests in turtles (Janzen, 1993; Janzen et al., 2000a, b, 2007, Tucker, 2000; Myers et al., 2007; Paitz et al., 2007; Tucker et al., 2008; but see Congdon et al., 1999; Kolbe and Janzen, 2001; Filoramo and Janzen, 2002). Such effects could confound inference in this study, particularly if they operate via the exposure time mechanism proposed above. Therefore, we also analyzed the effect of body size (CL and mass) on survival during dispersal using logistic regression. To explicitly test the exposure time link between body size and survival, we performed linear regressions using body size measures to predict dispersal times. Clutch effects were not analyzed statistically in any of our models due to sample size limitations. Instead, the impact of clutch in each analysis was assessed qualitatively by identifying clutches in which mortality was high regardless of treatment or sex. Descriptive statistics were computed using JMP 5.1.2 (SAS Institute Inc, 2004) and regression analyses were performed using SAS 9.1.3 (SAS Institute Inc, 2003).

## Results

Of the 144 eggs incubated, 129 successfully hatched (89.6%; Table 2) and success ranged by treatment from 73.3% (30CNM) to 100% (30NM; Table 2). Female-producing temperatures resulted in somewhat lower success than did male-producing temperatures (85.2 vs. 92.2%, respectively; χ2_1_ = 1.7, *p* = 0.19) but this was heavily influenced by one control clutch (30CM/30CNM) in which five of six eggs failed.

**TABLE 2:** Hatching success and post-hatching survival as a function of treatment and sex.

Treatment	Sex	Hatch	Winter	Dispersal	Total
*26NM*	M	21/24 (0.875)	19/21 (0.905)	13/19 (0.684)	13/24 (0.542)
30M	M	23/24 (0.958)	21/23 (0.913)	11/21 (0.523)	11/24 (0.458)
26CNM	M	19/21 (0.905)	19/19 (1.0)	12/19 (0.667)	12/21 (0.571)
26CM	M	20/21 (0.952)	20/20 (1.0)	12/17 (0.706)	12/21 (0.571)
30NM	F	12/12 (1.0)	12/12 (1.0)	7/12 (0.583)	7/12 (0.583)
*26M*	F	11/12 (0.917)	11/11 (1.0)	10/11 (0.909)	10/12 (0.833)
30CNM	F	11/15 (0.733)	10/11 (0.909)	4/10 (0.4)	4/15 (0.267)
30CM	F	12/15 (0.8)	9/12 (0.75)	0/9 (0.0)	0/15 (0.0)
	M	83/90 (0.922)	79/83 (0.952)	48/75* (0.640)	48/90 (0.533)
	F	46/54 (0.852)	42/46 (0.913)	21/42 (0.50)	21/54 (0.389)
Total		129/144 (0.896)	121/129 (0.958)	69/118 (0.585)	69/144 (0.479)

Success and survival are reported as the fraction (proportion) of individuals hatched or alive at the end of a given stage relative to the number in existence at the start of that stage. The first four rows correspond to treatments in the catch-up experiment of Colbert et al. (2010); treatment abbreviations are: hatchlings initially incubated at 30°C and moved to 26°C (30M), hatchlings initially incubated at 26°C and not moved (*26NM*), control hatchlings initially incubated at 26°C and moved to 26°C (26CM), and control hatchlings initially incubated at 26°C and not moved (26CNM). The next four rows correspond to treatments in the wait experiment of Colbert et al. (2010); treatment abbreviations are: hatchlings initially incubated at 26°C and moved to 30°C (*26M*), hatchlings initially incubated at 30°C and not moved (30NM), control hatchlings initially incubated at 30°C and moved to 30°C (30CM), and control hatchlings initially incubated at 30°C and not moved (30CNM). The two treatments hypothesized to result in reduced survivorship are italicized. * indicates one turtle failed to depart its nesticle and is excluded here from accounting.

**FIGURE 2:**
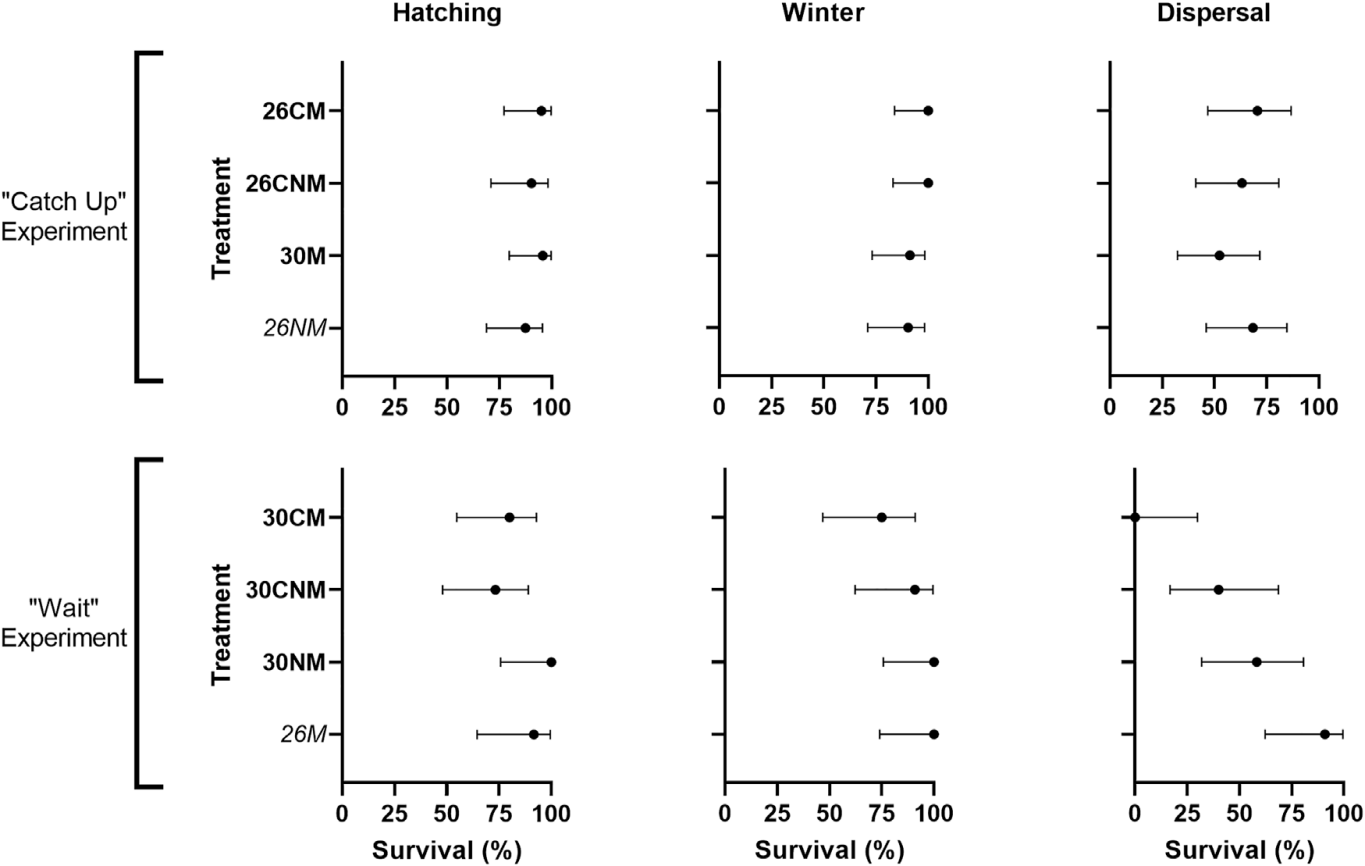
Hatching success and survival of early-hatching embryos versus their corresponding controls and/or sibs (top) early-hatching turtles from the catch-up experiment (*26M*), their controls (26CM), and sibs (30NM) and (bottom) suspected early-hatching turtles from the wait treatment (*26NM*) and their sibs (30M). Hatching success and post-hatching survival are reported as the proportion of individuals hatched or alive at the end of a given stage relative to the number in existence at the start of that stage, expressed as a percent. 95% confidence intervals are calculated using the Wilson/Brown method in Prism software (v9.4.0, GraphPad Software). The two treatments expected to suffer a cost are italicized.

Treatment-specific contrasts were consistent with our predictions; early-hatching groups suffered reduced hatching success when compared to their controls and/or sibs (Figure 2). Yet, among all groups, early-hatching treatments (26M and 26NM) exhibited intermediate hatching success, and incubation treatment was not a statistically significant source of variation (χ2_7_ = 6.0, *p* = 0.54). The high mortality in the single control clutch noted above did not account for lack of model fit, as exclusion of this clutch did not alter results (χ2_7_ = 3.4, *p* = 0.85).

Winter survival was high overall; 121 of the 129 turtles that hatched successfully were alive in the spring (93.8%; Table 2). Survival was lowest in the 30CM treatment (75%), and several treatments suffered no losses over winter (Table 2). Sex did not influence winter survival (χ2_1_ = 0.7, *p* = 0.39), with males and females having similar high survival rates (91.3% female vs. 95.2% male). Clutch was an important factor in winter survival for both sexes; male and female groups each had a single clutch that disproportionately reduced overall survival (50% mortality in both cases).

Our predictions with respect to treatment were not supported. No reduction in winter survival was apparent for the early-hatching treatments (Figure 2), nor did incubation regime significantly affect winter survival (χ2_7_ = 2.2, *p* = 0.95). The clutch effect noted above likely had little effect on treatment-specific survival, as mortality was distributed near evenly over four treatment groups (30CM, 30CNM, 26NM, and 30M).

Apparent survival during terrestrial dispersal averaged 58.5% over all treatments (Table 2). One individual failed to disperse and was recovered alive from its nesticle at the end of the experiment (excluded from analyses). The distribution of recovery sites (i.e., pit numbers) was approximately normal with a mean value of 3.75, which was near the center of the fence (pit 4). No individuals were recovered near the terminal pits (1 and 7), and the 99% confidence interval about the mean pit value was narrow and nearly centered (3.4–4.1). Thus, it is unlikely that any neonates escaped recapture by missing the fence. The final fence check occurred 5 days after the last individual was recaptured, and no turtles (alive or dead) were found in the release area during a thorough inspection at the end of the experiment. Therefore, it is also unlikely that turtles were migrating at the end of the experiment and recorded as false mortalities. Consequently, 58.5% should be a close approximation to true survival, and failure to find carcasses within the release area points to predation as the primary source of mortality. Potential predators observed in the release site included brown thrasher (*Toxostoma rufum*), common grackle (*Quiscalus quiscula*), red-winged blackbird (*Agelaius phoeniceus*), blue racer (*Coluber constrictor*), and a coyote (*Canis latrans*) pup.

Treatment-specific survival ranged from 0 (30CM) to 90.9% (26M). Females exhibited reduced survival relative to males, but this difference was not statistically significant (50.0 vs. 64.0%, respectively; χ2_1_ = 1.7, *p* = 0.20). Although the magnitude of clutch effects during migration and overall was not discerned, only two clutches were not represented among the survivors.

Our hypothesis that reduced incubation period would lead to lower survival during migration was not supported. Rather, early hatching modestly increased survivorship (Figure 2; Table 2), however, such differences were not statistically significant (χ2_7_ = 6.7, *p* = 0.47).

Individual dispersal times ranged from 0.25–8.75 days, and treatment averages from 1.1–3.5 days. The distribution of recapture times was right-skewed with a mean recapture time of 1.7 days (99% CI = 1.2–2.2 days). Sex effects were not apparent, as males and females required approximately the same amount of time to reach the fence (1.75 and 1.95 days, respectively; *F*_1_ = 0.2, *p* = 0.68). Similarly, clutch did not appear to affect dispersal as, within clutches, times generally ranged the entire spectrum from short (<1.2 days) to long (>2.2 days).

**FIGURE 3:**
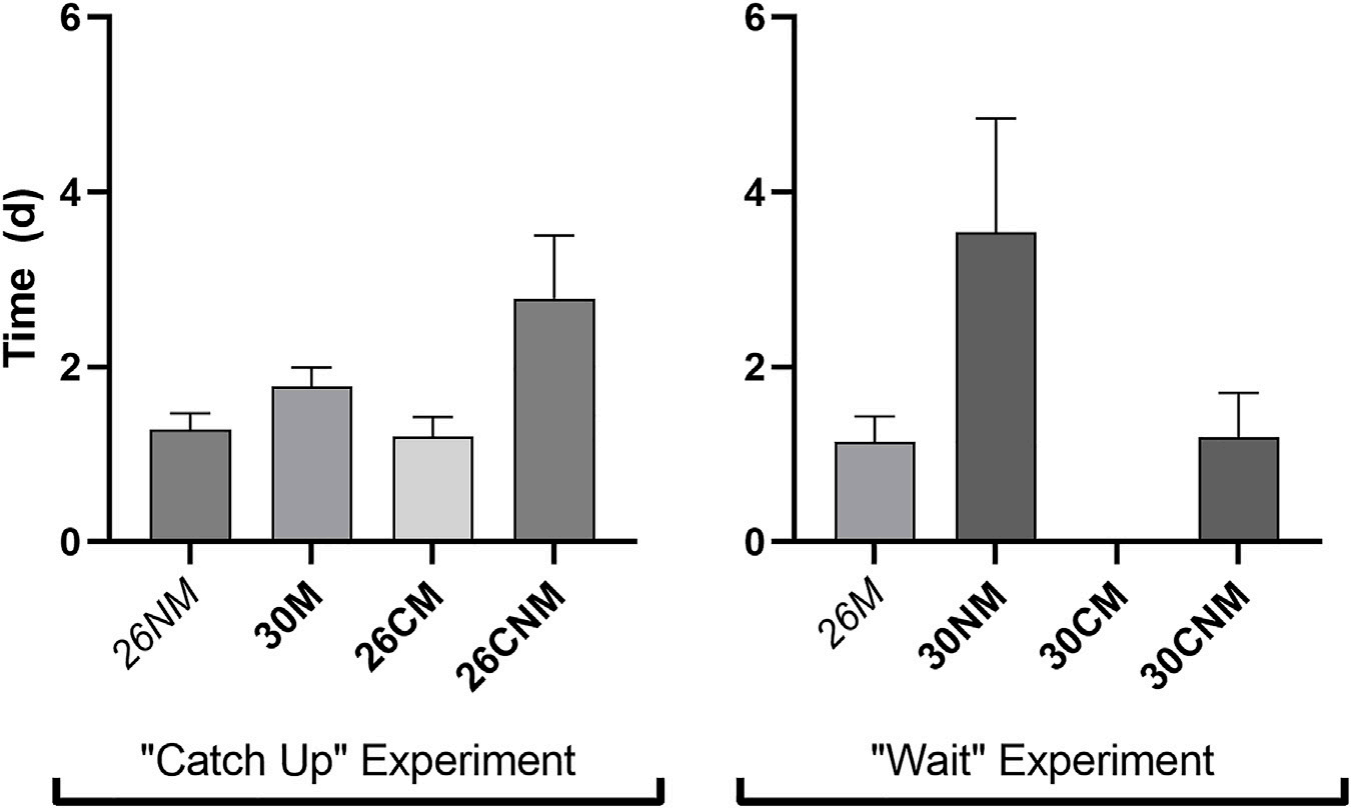
Time (d) required to reach the drift fence for early-hatching embryos versus their corresponding controls and/or sibs. The two early-hatching treatments in the two experiments expected to require the most time to disperse are italicized.

Incubation regime impacted the amount of time required for neonates to reach the fence (*F*_6_ = 2.8, *p* = 0.02). However, contrary to expectation, early-hatching groups were among the fastest relative to both sibs and/or controls (Figure 3) and overall. The average travel time for the 26M group (1.1 d) was below the lower bound of the 99% CI about mean recapture time, and considerably shorter than that of their sibs (30NM, *F*_1_ = 8.6, *p* = 0.005; Figure 3). Consistent with the exposure time hypothesis, the group with the shortest travel time also exhibited the highest survivorship during terrestrial dispersal (26M). However, the two treatments that averaged longer than 2.2 days to reach the fence (26CNM and 30NM) had intermediate survivorship.

Mean measures of CL and body mass of all individuals released were 27.2 mm (SD ± 1.6 mm, range = 24.0–31.3 mm) and 4.55 g (SD ± 0.7 g, range = 2.82–6.01 g) and neither measure differed between sexes (*F*_1_ = 0.5, *p* = 0.47 and *F*_1_ = 2.7, *p* = 0.10, respectively). Results from logistic regressions indicated that neither size measure had a meaningful impact on survival during dispersal (CL: χ2_1_ = 0.4, *p* = 0.53; mass: χ2_1_ = 0.04, *p* = 0.85), even though inverse relationships existed between CL and dispersal time (*r* = -0.27, *F*_1_ = 5.3, *p* = 0.02) and between mass and dispersal time (*r* = -0.23, *F*_1_ = 3.8, *p* = 0.06).

## Discussion

We expected that premature hatching and its associated decline in performance (Colbert et al., 2010) would translate into reduced survival at hatching, during over-wintering, and during terrestrial dispersal, but we detected no such fitness costs in this study. Although not statistically significant, the pattern of hatching success was consistent with our predictions (Figure 2) and Colbert et al. (2010) reported that turtles that hatched prematurely had increased volumes of protruding yolk sacs. Riley et al. (2020) did not find that latter pattern in the two turtle species they investigated, however. Evidence from birds also yields conflicting results (Nilsson and Persson 2004). Early-hatching painted turtles may have reduced capacity to right themselves, but they were among the fastest to complete dispersal in this study. Similarly, Irschick et al. (2005) found a general inverse relationship between maximum sprint speed in the laboratory and the percentage of maximum speed used to escape threats in the field in lizards. The ability for a turtle to right itself quickly does not necessarily reduce predation risk (Mitchell et al., 2016; but see Delmas et al., 2007).

Our overwintering temperatures did not necessarily expose turtles to extreme cold (5°C), but such conditions may represent future overwintering temperature regimes as climates warm. Indeed, winter temperatures in the contiguous U.S. already have risen 3°C in the past 125 years (USGCRP et al., 2017). Minimum temperatures in *C. picta* nests from our study population in Illinois during the winter of 1995–1996 ranged from approximately −12 to −2°C (Weisrock and Janzen, 1999), yet only fell below −8°C in one nest in the winter of 2016–2017 (Murphy et al., 2020). On the other hand, above-freezing temperatures impose significant metabolic demands on neonatal turtles compared to below-freezing conditions (Willette et al., 2005), where metabolic rates are negligible (Spencer and Janzen, 2011). Hence, although this experiment did not strongly test the cold tolerance of early-hatching turtles, it suggests they can meet increased metabolic burdens.

Although we found no evidence for sex effects on hatching success and post-hatching survival in this study, we see a cumulative reduction in fitness for females across all three stages (Table 2). Sex-specific differences in survival have been documented in other turtle species (e.g., Steyermark and Spotila, 2001b; Freedberg et al., 2001, 2004), as well as a range of other traits in painted turtles (Warner et al., 2020). In species with TSD, sex and temperature effects on life-history traits are often confounded, and the absence of statistically significant sex effects on survival at each stage in this study may be due to the thermal variance incorporated through temperature switches, which could have produced intermediate phenotypes and thus blurred group distinctions. Comparisons of control clutches only (26CM and 26CNM vs. 30CM and 30CNM), which experienced no thermal variance, show that cooler incubation temperatures resulted in higher success and survivorship at every stage considered (Table 2), significantly so over winter (χ2_3_ = 9.46, *p* = 0.024) and during terrestrial dispersal (χ2_3_ = 15.9, *p* = 0.001). Because sex-specific differences were only apparent when incubation temperatures were held constant, this finding supports the hypothesis that such differences in early life are attributable primarily to incubation temperatures. Hence, long exposure to constant temperatures may induce sex effects and may not reflect temperatures in nature, where constant temperatures are rare, particularly in shallow nests of freshwater turtles (Thompson, 1988; Weisrock and Janzen, 1999).

In this study, body size did not influence survival during dispersal contra Paitz et al. (2007) for the same population, although we found partial support for reduced exposure times of larger turtles. The interaction of numerous dynamic biotic and abiotic factors likely results in varying selection on offspring traits such that no single factor consistently predicts dispersal success (e.g., Marshall et al., 2006). When predation is high, body size effects may be more pronounced (e.g., Janzen et al., 2000a, b), whereas dry conditions may force young turtles to burrow for extended periods, altering the landscape of selective pressures (e.g., Kolbe and Janzen, 2002b; Filoramo and Janzen, 2002). At other sites or in other years, microhabitat characteristics such as vegetation density and slope may modify the relationship between offspring traits and survival (e.g., Kolbe and Janzen, 2001). Discrepancies in experimental studies of phenotypic selection during early-life dispersal indicate that there may be no straightforward relationships. Although our study reveals no effect of treatment, sex, or body size, the incubation conditions and post-hatching handling of turtles in the laboratory or field may help to explain the lack of consistent patterns and variability between studies. The ontogeny of neonatal turtles in the nest is likely to encompass a complex interaction between genetics, maternal effects, sex, incubation temperature, hatching date, and post-hatching environmental conditions, making general comparisons difficult between species and populations.

This study provides insight into the evolution and maintenance of hatching synchrony in turtles, yet cannot strongly refute either the multiple origins or phylogenetic inertia interpretation of Colbert et al. (2010). Consistent with the inertia hypothesis, our data reject that shortening development time to hatch synchronously reduces hatching success. In addition, we found no evidence that reduced performance in the laboratory translated into reduced fitness in a simulated post-hatching nest environment or during terrestrial dispersal. Finally, although the distribution of hatching synchrony in turtles is poorly understood, the trait has been identified in multiple families and both megaorders of turtles, lending support to a single, basal origin of this phenomenon. Future work should focus on hatching synchrony in other turtle taxa to more firmly resolve these two hypotheses in a formal phylogenetic comparative analysis.

The consequences of environmentally-induced hatching more generally constitute an emerging field of research (Sih and Moore, 1993; Blaustein, 1997; Warkentin, 2005; Doody, 2011; Spencer and Janzen, 2011). Our experiment is novel in linking elements of physiology, ecology, and evolution to elucidate proximate and ultimate functions of synchronous hatching. As further studies are conducted on diverse taxa, we not only will better elucidate evolutionary costs and benefits but also will gain greater insight into physiological mechanisms and ecological dynamics, ultimately leading to a broader understanding of environmentally-induced traits.

## Data Availability

The raw data supporting the conclusions of this article will be made available by the authors, without undue reservation.
